# Thermophysiological comfort of zinc oxide nanoparticles coated woven fabrics

**DOI:** 10.1038/s41598-020-78305-2

**Published:** 2020-12-03

**Authors:** Muhammad Tayyab Noman, Michal Petru, Nesrine Amor, Petr Louda

**Affiliations:** 1 Department of Machinery Construction, Institute for Nanomaterials, Advanced Technologies and Innovation (CXI), Technical University of Liberec, 461 17, Studentská 1402/2 Liberec 1, Czech Republic; 2 Acoustic Signal Analysis and Processing Group, Faculty of Mechatronics, Informatics and Interdisciplinary Studies, Technical University of Liberec, 461 17, Studentská 1402/2 Liberec 1, Czech Republic; 3 Department of Material Science, Faculty of Mechanical Engineering, Technical University of Liberec, 461 17, Studentská 1402/2 Liberec 1, Czech Republic

**Keywords:** Materials science, Nanoscience and technology

## Abstract

This study investigates physicochemical impact of ultrasonic irradiations on surface topography of woven fabrics. In a simultaneous in-situ sonochemical method, the synthesis and coating of zinc oxide nanoparticles (ZnO NPs) on woven textiles were successfully achieved. Different instruments i.e. Alambeta, moisture management tester, air permeability tester and permetester were utilised during experimentation for thermal evaluation, moisture transportation and air permeation. The results regarding thermophysiological comfort of ZnO coated fabrics were evaluated on the basis of thickness and ZnO NPs coated amount on fabrics. In addition, the achieved results depict the impact of sonication (pressure gradient) on surface roughness of cotton and polyester. The coating of ZnO NPs on fabrics, crystal phase identification, surface topography and fluctuations in surface roughness were estimated by inductively coupled plasma atomic emission spectroscopy (ICP-AES), X-ray Diffractometry (XRD), ultrahigh-resolution scanning electron microscopy (UHR-SEM) and energy dispersive X-ray (EDX). Moreover, thermophysiological properties i.e. thermal conductivity, absolute evaporative resistance, thermal absorptivity, air permeability, overall moisture management capacity and relative water vapour permeability of untreated and ZnO treated samples were evaluated by standard test methods.

## Introduction

In textiles, thermophysiological comfort is considered as most demanding and desirable characteristics that is achieved by maintaining heat and mass transfer phenomena. Comfort helps the customer to choose a suitable fabric for cold as well as for hot weather. From experimental point of view, thermophysiological and sensorial comfort are two most important and significant categories of clothing comfort among other categories. In recent years, many researchers worked with different textiles for thermophysiological comfort and reported interesting results but their results were based on thermophysiological properties of plain non-coated fabrics (nanostructures were not applied on samples)^1–3^. Azeem et al. studied thermophysiological properties of multifilament polyester non-coated fabric and claimed that nanofilament fabric possess remarkably higher thermal conductivity than coolmax and pure cotton. Their work composed of non-coated samples for all substrates. A lower value of thermal absorptivity induced warm feeling and a higher value gives cool feeling in nanofilament polyester fabric^4^. In another study, Angelova et al. proposed a comprehensive comfort analysis of woven and non-woven fabrics made of cotton, polyester and polyamide. Their work was also composed of non-coated samples for all kind of textile substrates. They reported that thermophysiological comfort is significantly affected by number of fabric layers, bulk density, fabric thickness and porosity of used materials^5^. Mishra et al. worked with comfort evaluation of 3D spacer knitted fabrics segregated into two major groups for a comprehensive analysis. They chose blended fabrics for both groups i.e. polyester/polypropylene and polyester/polypropylene/lycra with different compositions. They found that water vapour permeability is highly affected by the type of textile materials. In addition, air permeability depends on fabric density and fabric thickness^6^. However, there are very limited studies based on nanomaterials coated textiles and their thermophysiological comfort evaluation^7,8^. In previous studies, thermophysiological evaluation of nano TiO_2_ coated fabrics were carried out and interesting results were obtained^9,10^.

ZnO is a fascinating material with exceptional physicochemical properties. These properties (thermal conductivity, high electron mobility, high exciton binding energy, wide band gap) allow ZnO to function under infinite range of applications^11^. Researchers have reported the synthesis and deposition of ZnO nanostructures by sol–gel, hydrothermal and chemical vapours deposition methods on textiles and other substrates for photocatalytic, photovoltaic and functional applications^12–20^. These approaches are time consuming and most of them involve two step deposition of ZnO. However, the process of sonication (utilization of ultrasonic energy) for the fabrication and incorporation of a variety of nanostructures (organic, inorganic, metallic, non-metallic etc.) on textiles has become facile, economical and environmentally friendly method^21^. This approach works through the principle of acoustic cavitation i.e. generation of unstable bubbles in liquids that violently collapse and increase temperature and pressure up to 5000 K and 20 MPa with cooling rate of 10^10^ K s^−1^ respectively^22^.

The literature regarding one step coating of ZnO NPs on textiles for the evaluation of comfort properties is extremely rare and to the best of knowledge, the only work on thermophysiological properties of ZnO coated cotton was reported by Dal et al.^23^. They studied comfort properties of ZnO coated twill cotton to a limited level. In a recent work, ZnO NPs with pure wurtzite crystal structure were fabricated and coated on cotton fabric by sonication under optimised conditions^24^. However, comfort properties were not discussed. Based on above discussion, it was concluded that a simultaneous synthesis and coating of ZnO NPs on textiles (single step) via sonication is a novel approach for the investigation of thermophysiological comfort evaluation. Therefore, in this study, a correlation between the deposited amount of ZnO NPs on different textiles via sonication and comfort properties was drawn. This study explicitly demonstrates and elaborates the effects of ultrasonic irradiations as a potential tool to deposit ZnO NPs on cotton and polyester fabrics for thermophysiological comfort. The approach delineates here thereby opens up a new avenue towards other textile substrates and coating materials.

## Materials and methods

### Materials

Woven fabrics (plain weave) made of 100% pure cotton and polyester threads used in this work. All other chemicals i.e. ZnCl_2_, NaOH and C_2_H_5_OH were used as received from Sigma Aldrich for the synthesis ZnO NPs.

### Physical testing

Fabric samples were conditioned before testing at 20 ± 2 °C temperature and 65 ± 2% relative humidity (standard conditions) for 24 h according to ASTM D 1776–16 standard test procedure. ASTM D 3776 was used to calculate gram per square meter (GSM) or fabric mass. Fabric thickness was determined according to standard test method ASTM D 1777–96 (2019). In addition, Table 1 shows the detail of necessary parameters of all coded samples.

**Table 1:** The detail of important parameters of samples and ICP-AES results.

Sample ID	Composition (100%)	Yarn count (tex)	Yarn density (threads/inch)	ZnO NPs coated amount (ppm)	GSM (g m^−2^)	Thickness (mm)
F_1_	Cotton	22	74	–	110	0.25
F_2_	Cotton	22	74	581	115	0.31
F_3_	Cotton	22	74	1090	118	0.38
F_4_	Cotton	28	52	–	224	0.66
F_5_	Cotton	28	52	598	229	0.72
F_6_	Cotton	28	52	1110	233	0.77
F_7_	Polyester	20	80	–	118	0.32
F_8_	Polyester	20	80	493	124	0.36
F_9_	Polyester	20	80	1032	128	0.41
F_10_	Polyester	27	58	–	230	0.66
F_11_	Polyester	27	58	583	234	0.78
F_12_	Polyester	27	58	1096	238	0.84

### Synthesis and coating of ZnO NPs

**Figure 1:**
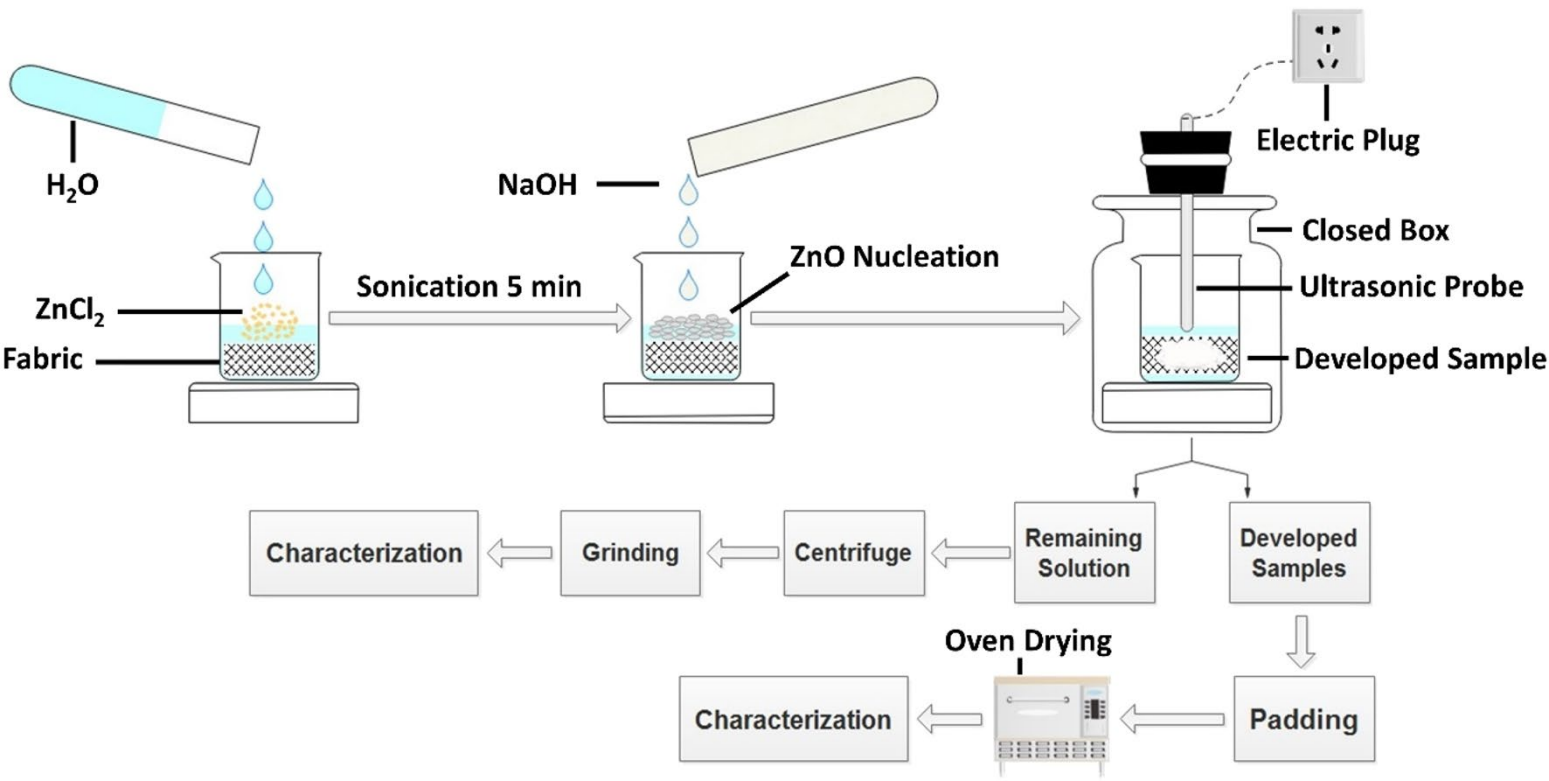
Graphical representation of proposed system and experimental study.

ZnO NPs were synthesized and coated on both fabric samples in a single step according to the procedure as described in previous investigation^22^. In a typical method, samples were immersed in a beaker and varying amount of ZnCl_2_ was added. Distilled water was added into the beaker to adjust 100 mL of total volume. The solution was sonicated for 5 min to prepare homogeneity. The granules of NaOH were added to solution in order to complete reaction. The running solution was sonicated for 1 h with ultrasonic probe homogeniser under optimised conditions. After sonication, samples were removed from their respective solution, squeezed on padder at a pressure of 3 kN with 1 m min^−1^ velocity and dried at 60 °C in an oven. The remaining solution of each sample was centrifuged at 4000 rpm to separate the flocculates of ZnO NPs from liquid for XRD analysis. The collected ZnO powders were dried at 80 °C for 1 h in oven to eliminate impurities and excess amount of moisture. A schematic explanation of the proposed method is presented in Fig. 1. ZnO NPs were characterised for crystal phase identification, morphology, topography and purity. SEM (UHR-SEM Zeiss Ultra Plus) was utilised to analyse morphological and topographical changes for all treated and untreated samples. EDX spectrophotometer was used to detect composition of elements and percentage amount of deposited materials. XRD analysis was performed for crystal phase identification. XRD patterns were matched with standard patterns of International Centre for Diffraction Data (ICDD) Powder Diffraction File (PDF: 89-7102). The coated amount of ZnO NPs on sample surface was calculated by ICP-AES analysis.

### Thermophysiological comfort properties

Alambeta instrument (developed by Sensora) was utilised for the evaluation of thermal conductivity coefficient (λ) (Wm^−1^ K^−1^) and thermal absorptivity (b) (Ws^1/2^ m^−2^ K^−1^). It also evaluates fabric thickness^9^. Alambeta calculates net amount of heat passes from a material having area of 1 m^2^ within 1 s and covers a distance of 1 m with temperature difference of 1 K. The coefficient of thermal conductivity is estimated by given equation.

1 $$\lambda = \frac{Q \times h}{{A \times t \times {\Delta T}}}$$

In Eq. (1), $Q$ shows net amount of heat flow, $h$ represents thickness, $A$ is cross-sectional area, $t$ is total time taken and $\Delta T$ shows temperature gradient respectively^9^.

Thermal absorptivity (b) is the measure of warm-cool feeling. Higher thermal absorptivity means cool feeling when body gets in touch with fabric and vice versa. The following equation calculates thermal absorptivity.

2 $$b = \sqrt {\rho \times \lambda \times c}$$

Permetester was utilised for relative water vapour permeability (RWVP) [%] and absolute evaporative resistance (R_et_) (m^2^ PaW^−1^). ISO 11092-2014 standard method was used to measure the values of RWVP and R_et_. Both parameters are useful in the evaluation of net water vapours transport capacity^9^. Equation (3) is used for the calculation of RWVP.

3 $$RWVP = \frac{{q_{f} }}{{q_{o} }} \times 100$$

In above equation, $q_{f}$ and $q_{o}$ represent heat loss with and without fabric sample respectively.

SDL ATLAS air permeability tester was utilised to determine air permeability by ISO 9237-1995 standard method. For this test, air pressure was set at 100 Pa^9^. Overall moisture management capacity (OMMC) is another vital attribute of thermophysiological comfort. Moisture management tester (MMT) was utilised for the measurement of OMMC. AATCC 195–2009 method was performed to determine OMMC. This property calculates the ability of textiles to deal with moisture^9^.

### Statistical analysis

The obtained results for all comfort related properties subjected to ZnO NPs coating by sonication method were statistically assessed by regression.

### Reusability

In order to confirm reusability and sequential performance, the durability of all treated samples was evaluated against washing by standard method ISO 105 C06. This method is considered as a direct approach to determine durability against washing. In this process, one cycle consists 4 g L^−1^ detergent that takes 45 min to complete the cycle at 50 °C. After samples removal, Zn^2+^ ions were calculated by ICP-AES. The study was repeated for five consecutive cycles.

## Results and discussions

### SEM and EDX analysis

**Figure 2:**
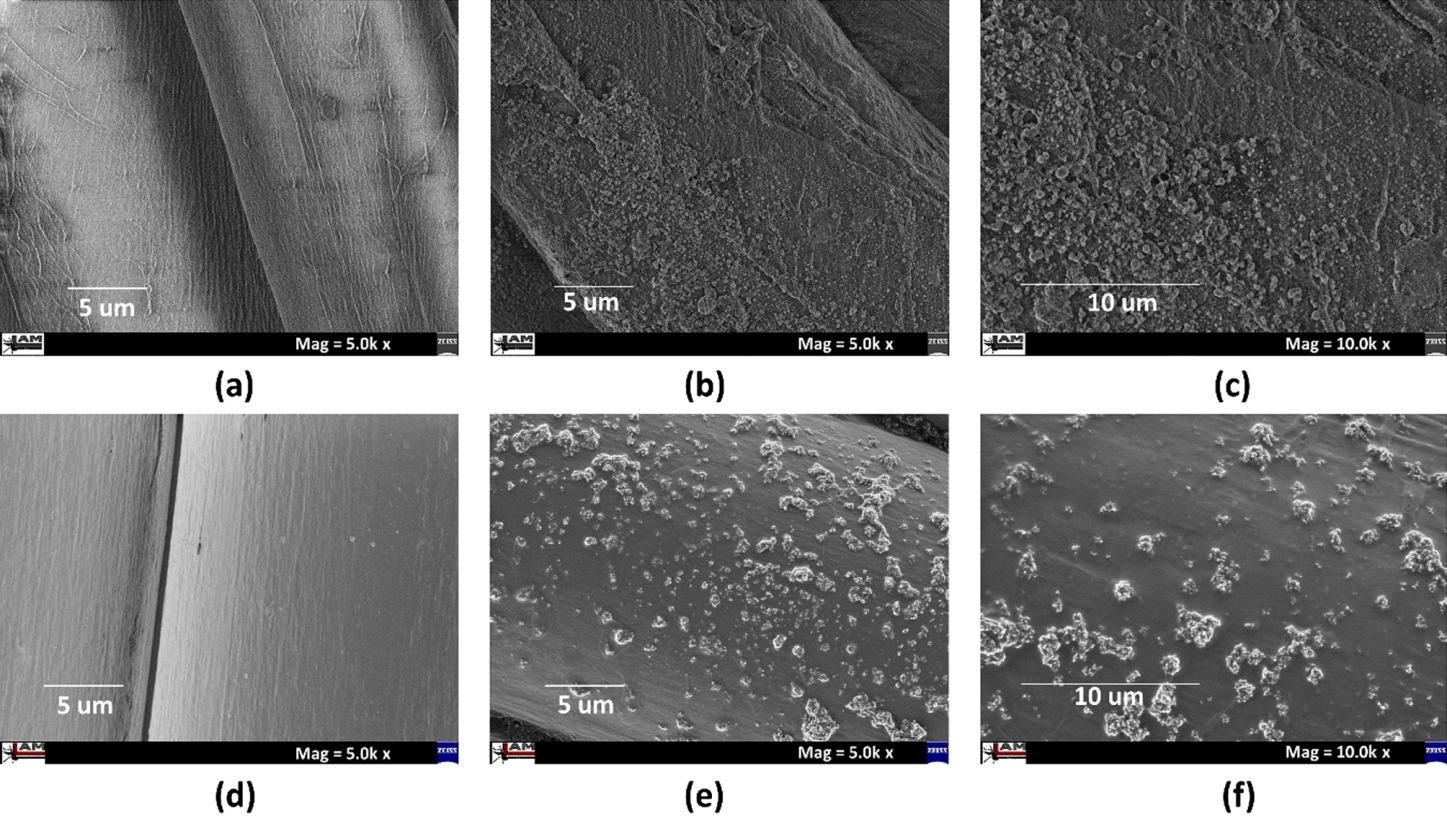
SEM images (**a**) untreated (**b**) sample F_3_ (**c**) sample F_3_ with higher magnification of cotton fabric and (**d**) untreated, (**e**) sample F_12_, (**f**) sample F_12_ with higher magnification of polyester fabric.

The results regarding morphology and surface topography of all coded samples are illustrated in Fig. 2. SEM images were carried out at 5.0 k and 10.0 k magnifications for cotton samples [F_1_ (untreated), F_3_ (treated)] and polyester samples [F_10_ (untreated), F_12_ (treated)]. A smooth and very clean surface of untreated sample of both substrates can be observed in Fig. 2a,d. Higher magnification was taken to visually guess estimated quantity (higher deposition or lower deposition) of ZnO NPs on treated fabrics. A homogenous distribution with quasi-spherical shape of ZnO NPs was detected for cotton whereas aggregation of particles was observed for polyester samples as depicted in Fig. 2b,c,e,f respectively. The impact of sonication was dominantly appeared in case of cotton as the surface was fully covered with ZnO NPs. However, the effects of sonication on polyester were appreciable and also acceptable.

**Figure 3:**
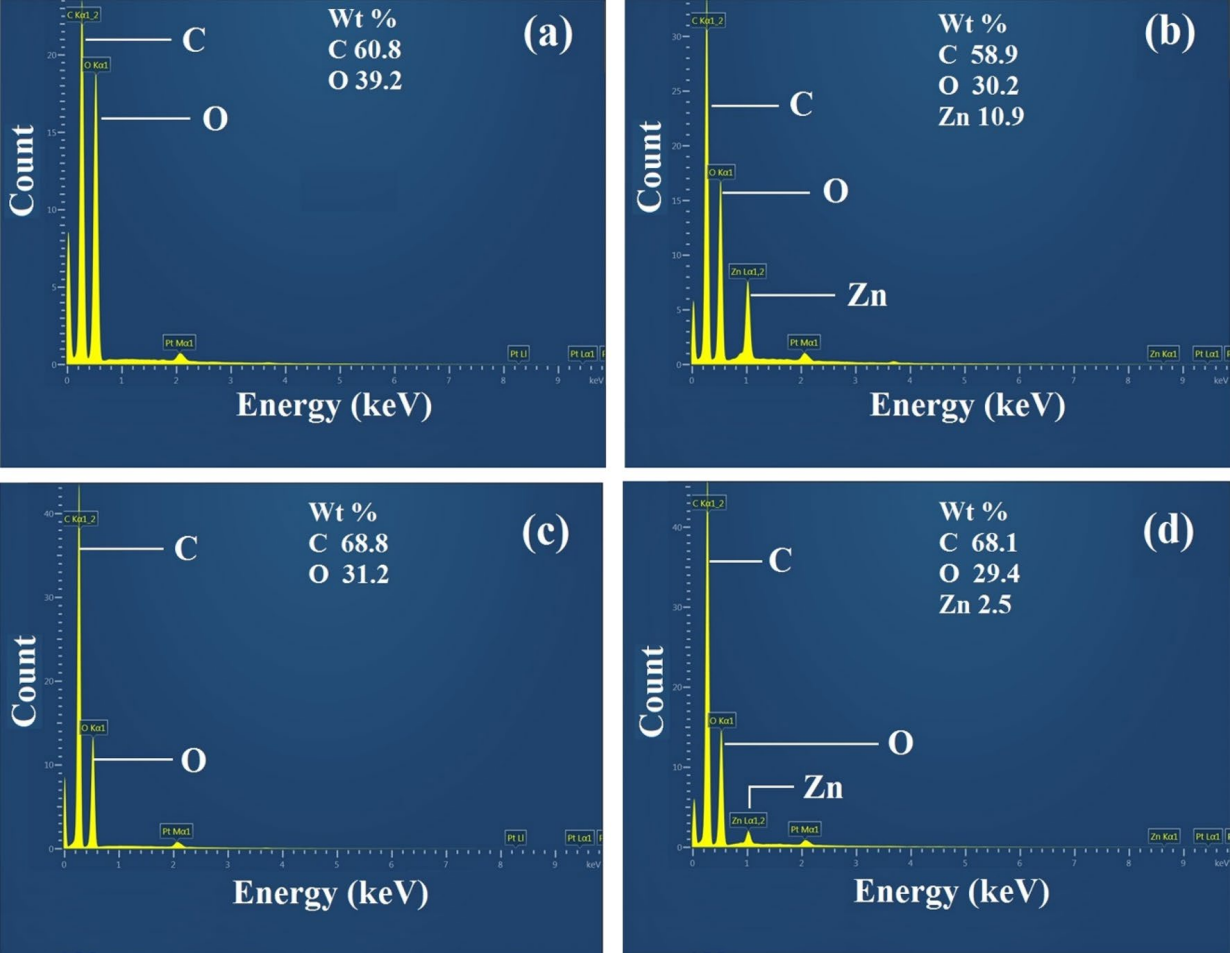
EDX spectra of cotton, sample F_1_ (**a**), sample F_3_ (**b**), and polyester, sample F_10_ (**c**), sample F_12_ (**d**) respectively.

Furthermore, EDX analysis was performed for the detection of elemental composition and weight percentage. The EDX results for sample F_1_, F_3_, F_10_ and F_12_ are presented in Fig. 3. EDX spectrum for samples F_3_ and F_12_ confirmed the existence of ZnO NPs on both fabrics as shown in (Fig. 3b,d). Though, Zn elemental peak was not found for untreated samples of cotton and polyester i.e. F_1_ (Fig. 3a) and F_10_ (Fig. 3c). In addition, EDX results confirm that the deposited amount of ZnO NPs was higher in cotton samples as compared to polyester. This happened due to different adsorption desorption phenomenon for cotton and polyester substrates. Due to ultrasonic energy, the surface roughness of cotton was higher as compared to polyester that ultimately results in more deposition of ZnO on cotton than polyester. The EDX results are in good agreement with SEM results as both techniques confirmed the excellent impact of sonication for cotton and good impact for polyester.

### XRD analysis

**Figure 4:**
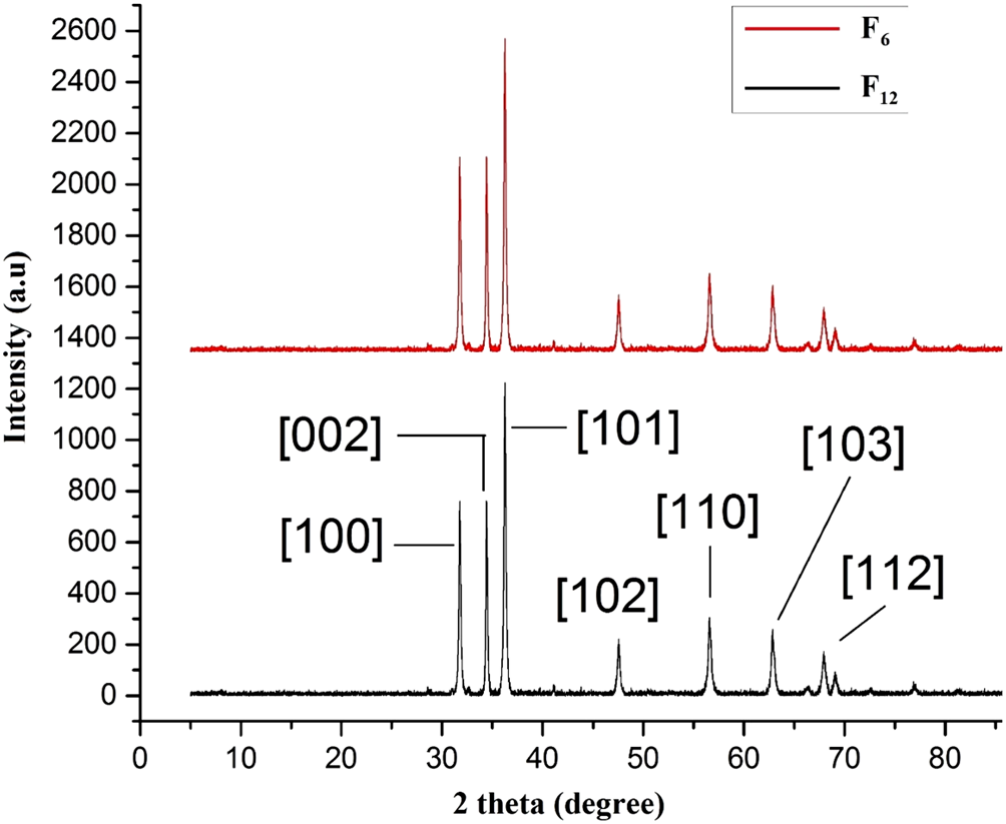
XRD results for samples F_6_ and F_12_.

XRD is a standard method used for identification of lattice structure and phase purity of a material. XRD results for samples (F_6_ and F_12_) confirmed wurtzite crystal structure of ZnO on both fabrics. The patterns show that all peaks matched with ICDD file (PDF: 89-7102). The highest peak appeared at 2θ = 36.2° is the characteristic peak of wurtzite that follows [101] plane reflection as illustrated in Fig. 4. In addition, a series of crystalline peaks at 2θ = 31.7°, 34.4°, 47.5°, 56.6°, 62.8° and 67.9° follow [100], [002], [102], [110], [103] and [112] planes respectively. Moreover, any phase other than ZnO called impurities i.e. Zn(OH)_2_ were not found.

### ICP-AES analysis

ICP-AES analysis confirmed the presence of ZnO NPs on both fabric samples. Though, ZnO was not present on untreated samples. Furthermore, for deposited amount of ZnO NPs on both substrates, elemental Zn peak was counted and results were presented in Table 1.

### Thermophysiological comfort analysis

The overall results of thermophysiological analysis of all samples with varied quantity of ZnO NPs are presented in Table 2 and discussed one by one here. This discussion is a description that comfort is a function of fabric thickness, ZnO NPs coated amount on fabric and ultrasonic irradiation time. In addition, regression statistics was performed to estimate influential tendency of selected variables on achieved responses.

**Table 2:** Thermophysiological properties of ZnO NPs coated fabrics.

Sample ID	Thermal conductivity (λ) (Wm^−1^ K^−1^)	Thermal absorptivity (b) (Ws^1/2^ m^−2^ K^−1^)	RWVP (%)	Absolute evaporative resistance (R_et_) (m^2^ pa W^−1^)	Air permeability (m^2^ s^−1^)	OMMC
F_1_	34.4	160	75.1	1.9	467	0.4512
F_2_	38.2	192	79.3	1.2	228	0.6241
F_3_	43.4	201	82.7	0.8	71	0.8104
F_4_	49.6	177	50.9	4.1	263	0.5021
F_5_	52.4	198	57.2	3.3	125	0.7132
F_6_	56.1	223	62.5	2.4	28	0.8954
F_7_	38.5	179	67.3	2.5	1189	0.3652
F_8_	42.7	191	71.8	1.9	882	0.7568
F_9_	47.3	200	76.1	1.3	624	0.8147
F_10_	48.2	186	47.5	5.3	712	0.3054
F_11_	53.7	199	56.2	3.6	465	0.4789
F_12_	59.2	219	68.4	1.7	341	0.7631

#### Thermal conductivity

**Figure 5:**
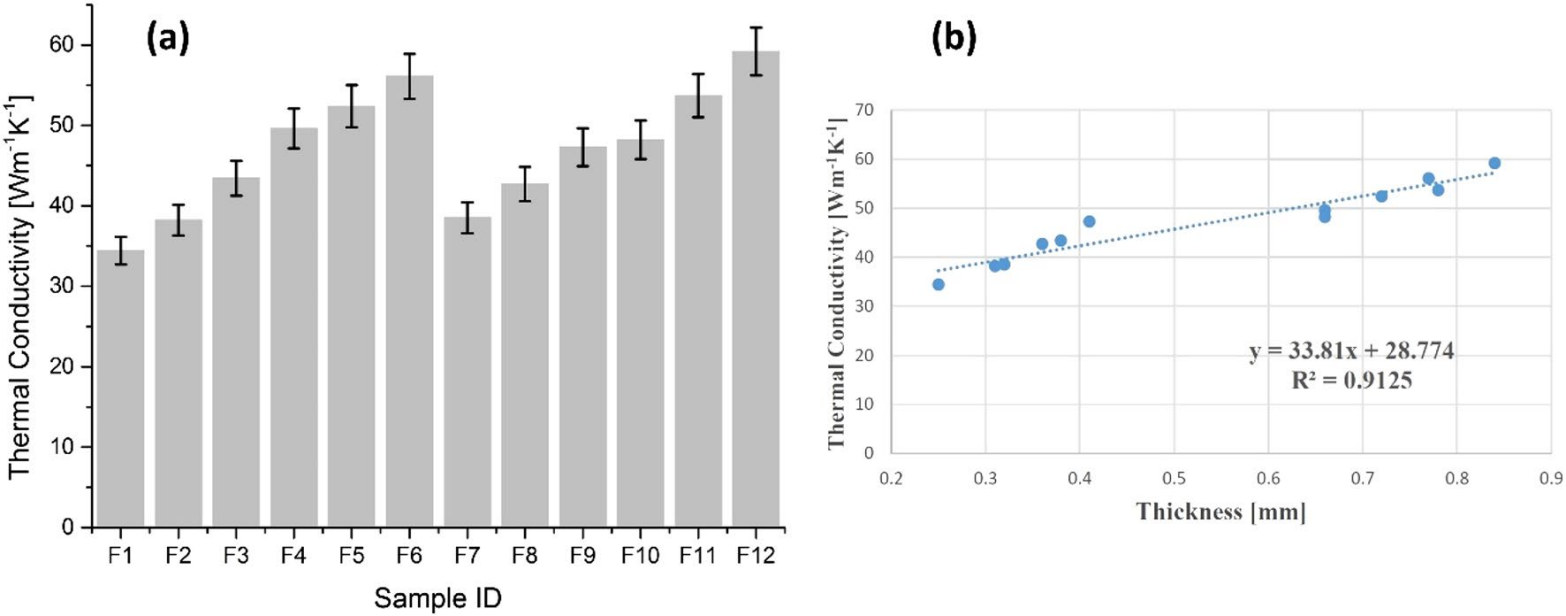
(**a**) Thermal conductivity of cotton samples (F_1_–F_6_) and polyester samples (F_7_–F_12_), and (**b**) thermal conductivity as a function of thickness.

Thermal conductivity is a significant and important approach to evaluate thermal comfort of any textiles. Thermal conductivity results are described in Fig. 5. Typically, higher thermal conductivity means more heat is transmitted from skin to fabric and ultimately generates cool feeling to body. This is an ideal situation for hot climate specifically in summer where thermal conductivity makes the process of heat transfer easy. The results were higher for all treated samples of cotton (F_2_, F_3_, F_5_, F_6_) and polyester (F_8_, F_9_, F_11_, F_12_) than untreated ones i.e. F_1_, F_4_ for cotton and F_7_, F_10_ for polyester (Fig. 5a). The results depict that ZnO coating on fabrics by sonication impart positive effects on porosity by covering the pores on fabric surface. ZnO NPs coating on both fabrics covered many empty spaces on fabric surface and reduced air entrapped inside fibre volume, and increased thermal conductivity. Moreover, higher ZnO NPs deposition increased fabric thickness and reduced the air portion in treated samples and eventually provided higher thermal conductivity. The observed results are in agreement with the findings of Dalbasi and Kayseri^25^.

Figure 5b explains the results of thermal conductivity as a function of thickness. For a better understanding, a typical point is provided that thickness itself is a function of ZnO NPs coated amount on fabrics that means thermophysiological properties are directly related to deposited amount of ZnO NPs on fabric. Therefore, comfort properties with a variation in thickness are elaborated and discussed. The trendline shows increasing tendency for thermal conductivity as thickness increased. Regression equation and R^2^ coefficient statically explain thermal conductivity dependency on sample thickness. A strong positive linear relationship and a strong dependency trend between fabric thickness and thermal conductivity was observed. Therefore, the developed products are a perfect option for summer wear. The results of thermal conductivity of ZnO NPs deposited woven fabrics (cotton, polyester) were slightly higher than TiO_2_ NPs deposited woven fabrics as reported in previous investigation^9^.

#### Thermal absorptivity

**Figure 6:**
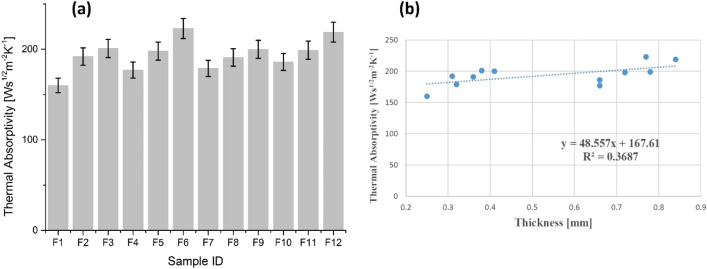
(**a**) Thermal absorptivity of cotton samples (F_1_–F_6_) and polyester samples (F_7_–F_12_), and (**b**) thermal absorptivity as a function of thickness.

Another important variable and a subject of great interest is thermal absorptivity in order to estimate warm-cool feeling. In general, lower thermal absorptivity means warm feeling and vice versa when fabric gets in touch with skin. The results of thermal absorptivity of all samples are shown in Fig. 6a. The results were higher for all treated samples of cotton (F_2_, F_3_, F_5_, F_6_) and polyester (F_8_, F_9_, F_11_, F_12_) than untreated samples i.e. F_1_, F_4_ for cotton and F_7_, F_10_ for polyester. The results showed that treated samples provide cool feeling. These results are reasonable as ZnO NPs coating on fabrics eliminated air gap among skin and fabric and increased total contact area. Therefore, higher values of thermal absorptivity were achieved as a result. The achieved results showed positive and significant impact of ZnO NPs coating on thermal absorptivity.

Figure 6b explains the results of thermal absorptivity as a function of thickness. The trendline shows an increased tendency of thermal absorptivity with an increase in fabric thickness. Regression equation and R^2^ coefficient statically explain thermal absorptivity dependency on sample thickness. A positive linear relationship with a dependency trend was observed between thermal absorptivity and thickness of fabrics. The achieved results are in agreement with the findings of Arumugam et al.^26^.

#### Relative water vapour permeability (RWVP)

**Figure 7:**
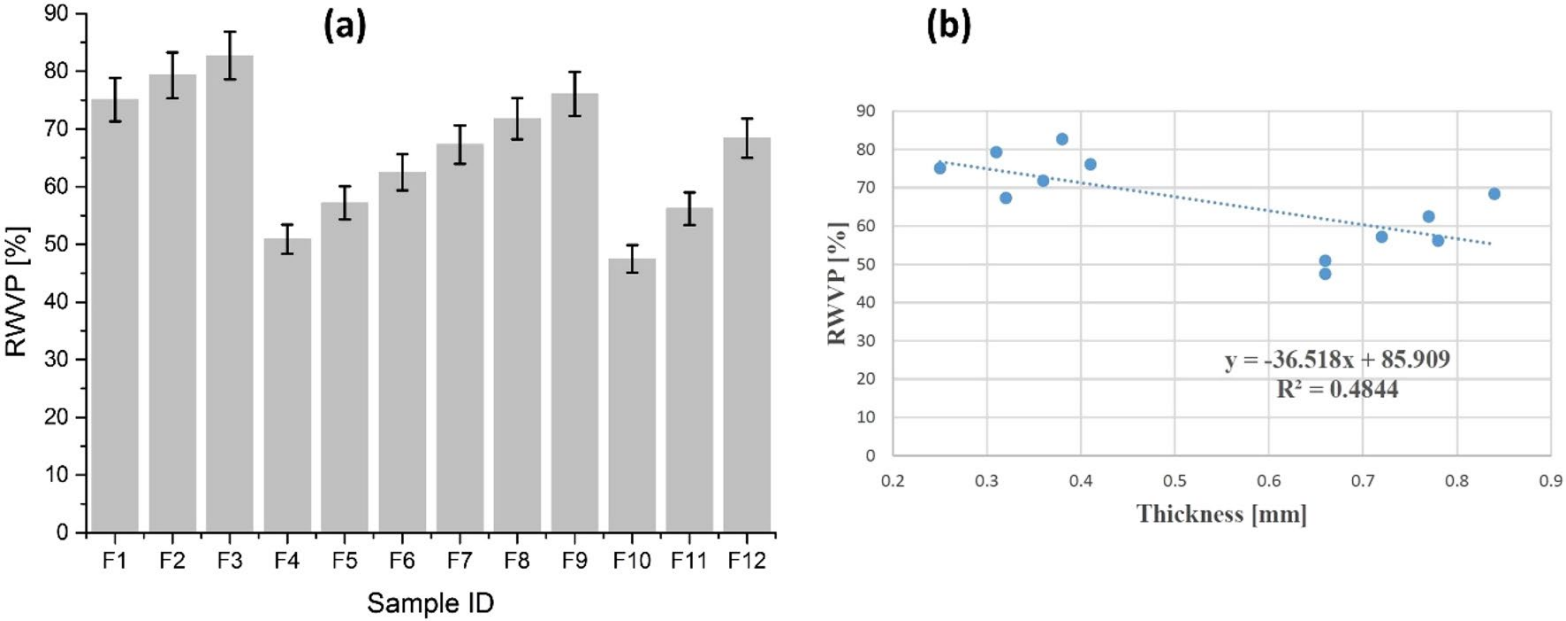
(**a**) Relative water vapour permeability of cotton samples (F_1_–F_6_) and polyester samples (F_7_–F_12_), and (**b**) relative water vapour permeability as a function of thickness.

The relative water vapour permeability is a non-standardized parameter that has a practical influence on overall thermophysiological comfort. The closer the value of RWVP of a substrate to 100 specifies more permeability or in other words, the substrate has excellent permeability. The results of RWVP are presented in Fig. 7a and the results explain that an augmentation in thickness reduced RWVP value for both fabrics. Moreover, an increase in mass per unit area of all samples leads a diminution in their respective RWVP value. However, a homogenous deposition of ZnO NPs with longer sonication time works as a carrier pathway for the transportation of water vapours that slightly enhanced RWVP for both type of substrates.

Figure 7b explains the overall results of RWVP as a function of thickness. The trendline shows a decreasing tendency of RWVP with an augmentation in thickness. The parameters of regression equation and the value of R^2^ coefficient statically explain the dependency of RWVP on material thickness. A negative linear relationship with a fair dependency trend was observed between thickness and RWVP performance of investigated samples. The obtained results are in agreement with the findings of Angelova et al.^5^.

#### Absolute evaporative resistance (R_et_)

**Figure 8:**
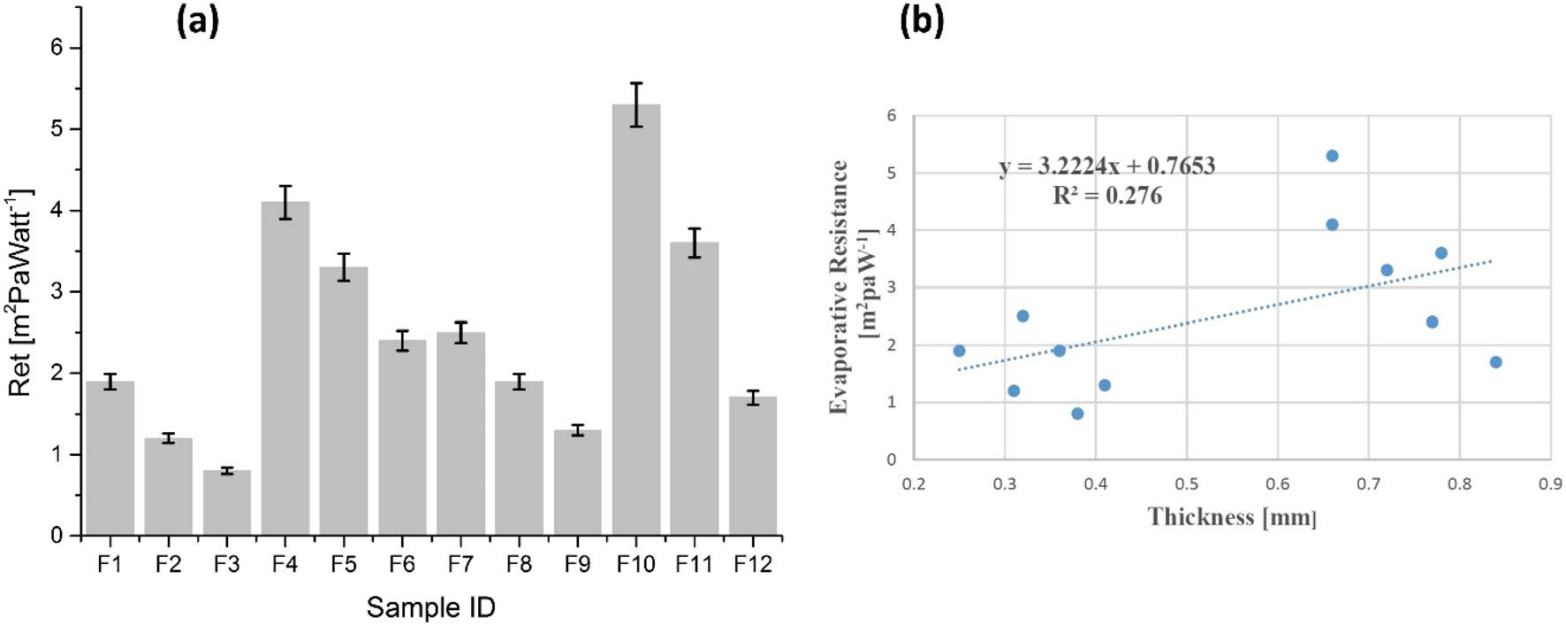
(**a**) Absolute evaporative resistance of cotton samples (F_1_–F_6_) and polyester samples (F_7_–F_12_), and (**b**) absolute evaporative resistance as a function of thickness.

Figure 8 shows the results of absolute evaporative resistance of all samples. The results depicted that an augmentation in fabric thickness enhances the evaporative resistance for observed samples. However, after the deposition of ZnO NPs via sonication method, a significant diminution was observed in absolute evaporative resistance (Fig. 8a). The obtained results further enlighten the benefits of sonication not only during the synthesis of novel materials but also for the augmentation of thermophysiological comfort.

Figure 8b depicts that absolute evaporative resistance is a function of thickness and the trendline shows an increased tendency with the augmentation of thickness. Regression equation and R^2^ coefficient statically explain absolute evaporative resistance dependency on fabric thickness. A positive linear relationship and a dependency trend was observed between evaporative resistance and sample thickness. The observed results are in good agreement with the findings of Zhou et al.^27^.

#### Air permeability

**Figure 9:**
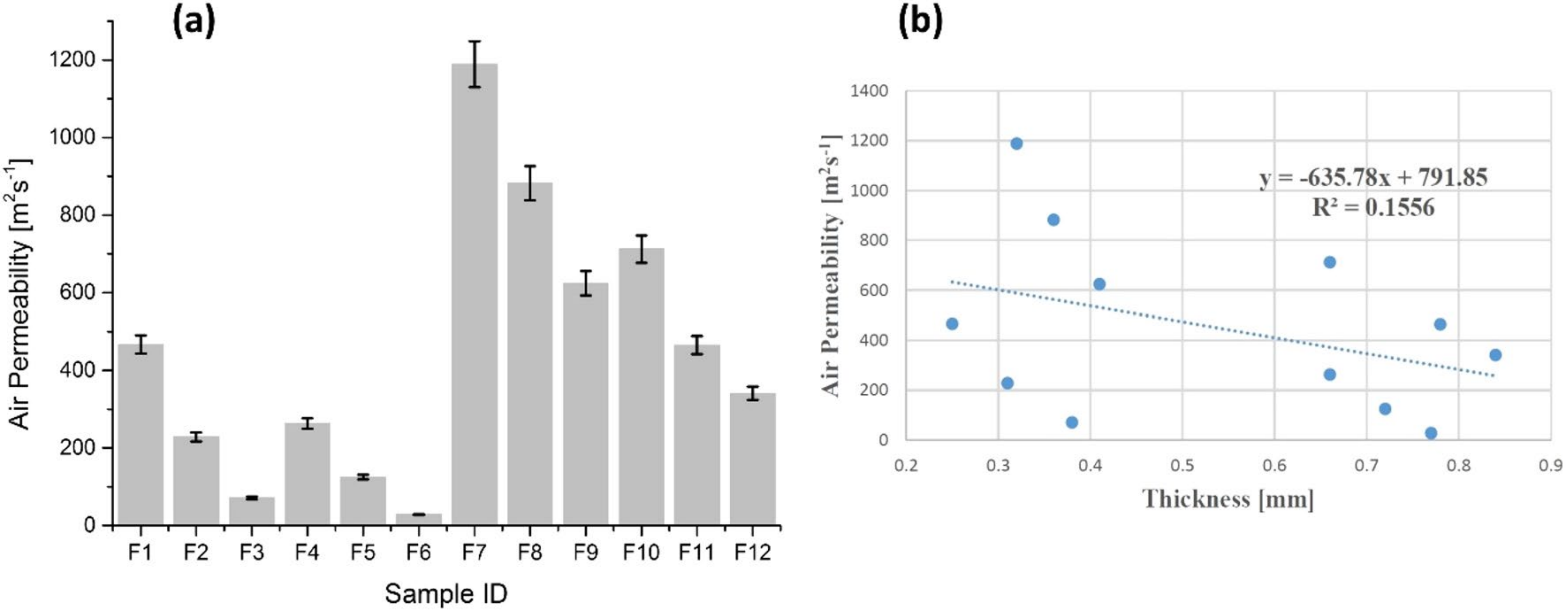
(**a**) Air permeability of cotton samples (F_1_–F_6_) and polyester samples (F_7_–F_12_), and (**b**) air permeability as a function of thickness.

Air permeability is another influential variable in order to evaluate thermophysiological comfort. This property performs an important role in moisture transportation from human body to external atmosphere. In principle, this property is based on pore size distribution by which air permeation takes place. Air permeability results are illustrated in Fig. 9a.

Air permeability values decreased significantly for all treated samples of cotton (F_2_, F_3_, F_5_, F_6_) and polyester (F_8_, F_9_, F_11_, F_12_) than untreated samples i.e. F_1_, F_4_ for cotton and F_7_, F_10_ for polyester. The results depicted that the deposition of ZnO NPs via sonication covers the portion of porosity by accumulating in void spaces and blocks the majority of pores. Additionally, ZnO NPs coating on fabrics induce difficulties in air pathway and results in the diminution of air permeability. These results are in good agreement with the findings of Shaid et al.^8^.

Figure 9b shows the results of air permeability as a function of thickness and trendline depicts a decreasing tendency of air permeability with the augmentation of thickness. In addition, Regression equation and R^2^ coefficient statically explain dependency of air permeability on sample thickness. A negative linear relationship and a random distribution was observed between air permeability and thickness.

#### OMMC

**Figure 10:**
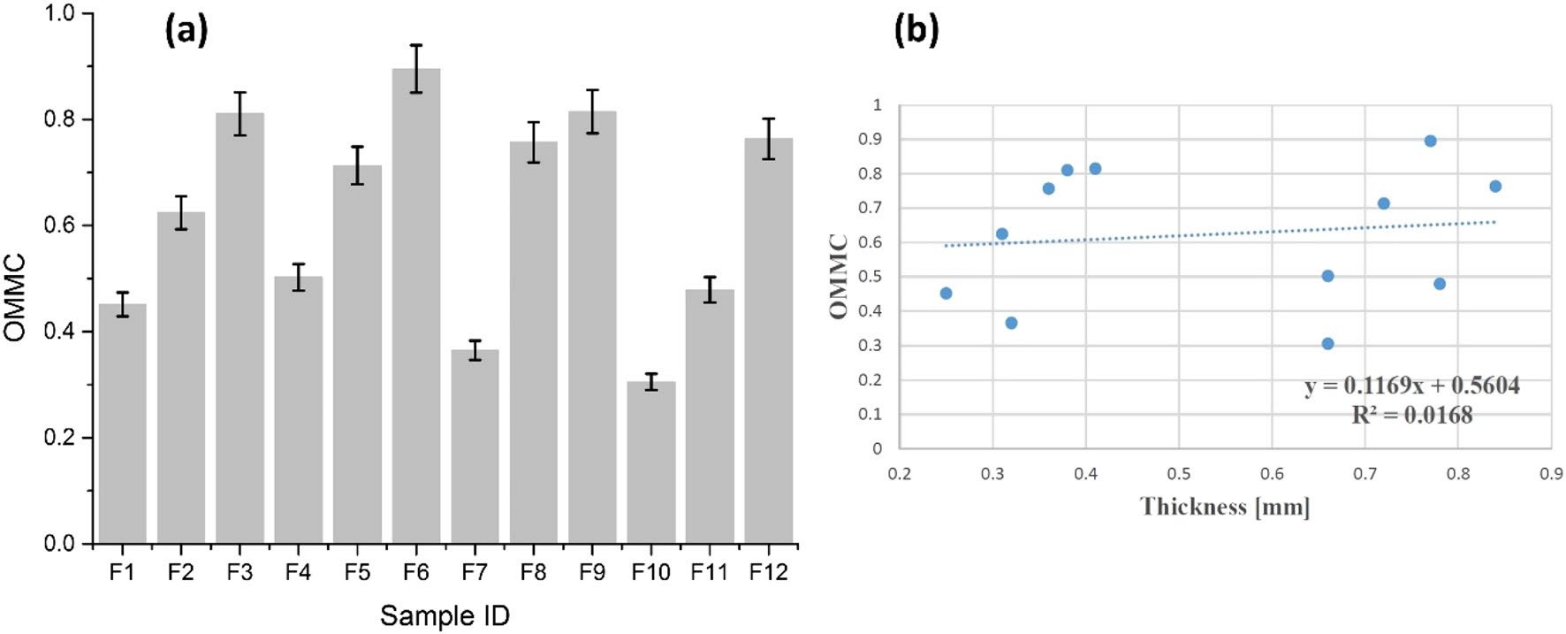
(**a**) The overall moisture management capacity of cotton samples (F_1_–F_6_) and polyester samples (F_7_–F_12_), and (**b**) the overall moisture management capacity as a function of thickness.

The OMMC is another important parameter and influential indicator for thermophysiological comfort evaluation. OMMC describes the capacity of a textile substrate to transfer liquid in all three dimensions. The results of OMMC of all samples are illustrated in Fig. 10a. The OMMC values ranges from (0 to 1) whereas a value closer to 1 indicates better moisture management properties of a substrate. OMMC results were higher for all treated samples of cotton (F_2_, F_3_, F_5_, F_6_) and polyester (F_8_, F_9_, F_11_, F_12_) than untreated samples i.e. F_1_, F_4_ for cotton and F_7_, F_10_ for polyester. The results show that the deposition of ZnO NPs through sonication induced positive and significant effects on the moisture management properties. In a previous study, the advantages of using sonication as an economical, user friendly and robust tool during the synthesis of nanomaterials and functional textiles is explained in details^28^. Fluid flow acceleration occurs inside fibre internal structure during sonication, and textile substrate swelling is achieved by acoustic cavitation phenomenon. These two factors resulted in better moisture management properties^29^. OMMC results are in agreement with the findings of Mishra et al.^6^.

Figure 10b presents the results of OMMC as a function of thickness and the trendline reveals a slight increase in OMMC values with an augmentation in thickness. Moreover, Regression equation and R^2^ coefficient statically explain OMMC dependency on sample thickness. A positive linear relationship with relatively random distribution was observed between OMMC and thickness.

**Figure 11:**
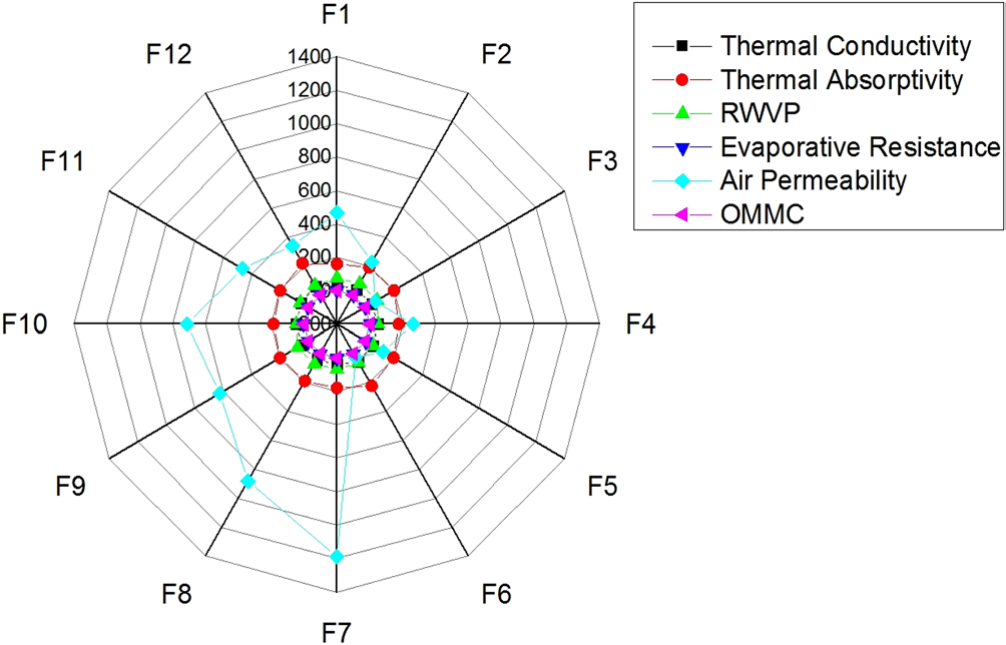
Spider plot of thermophysiological evaluation of used fabrics.

**Figure 12:**
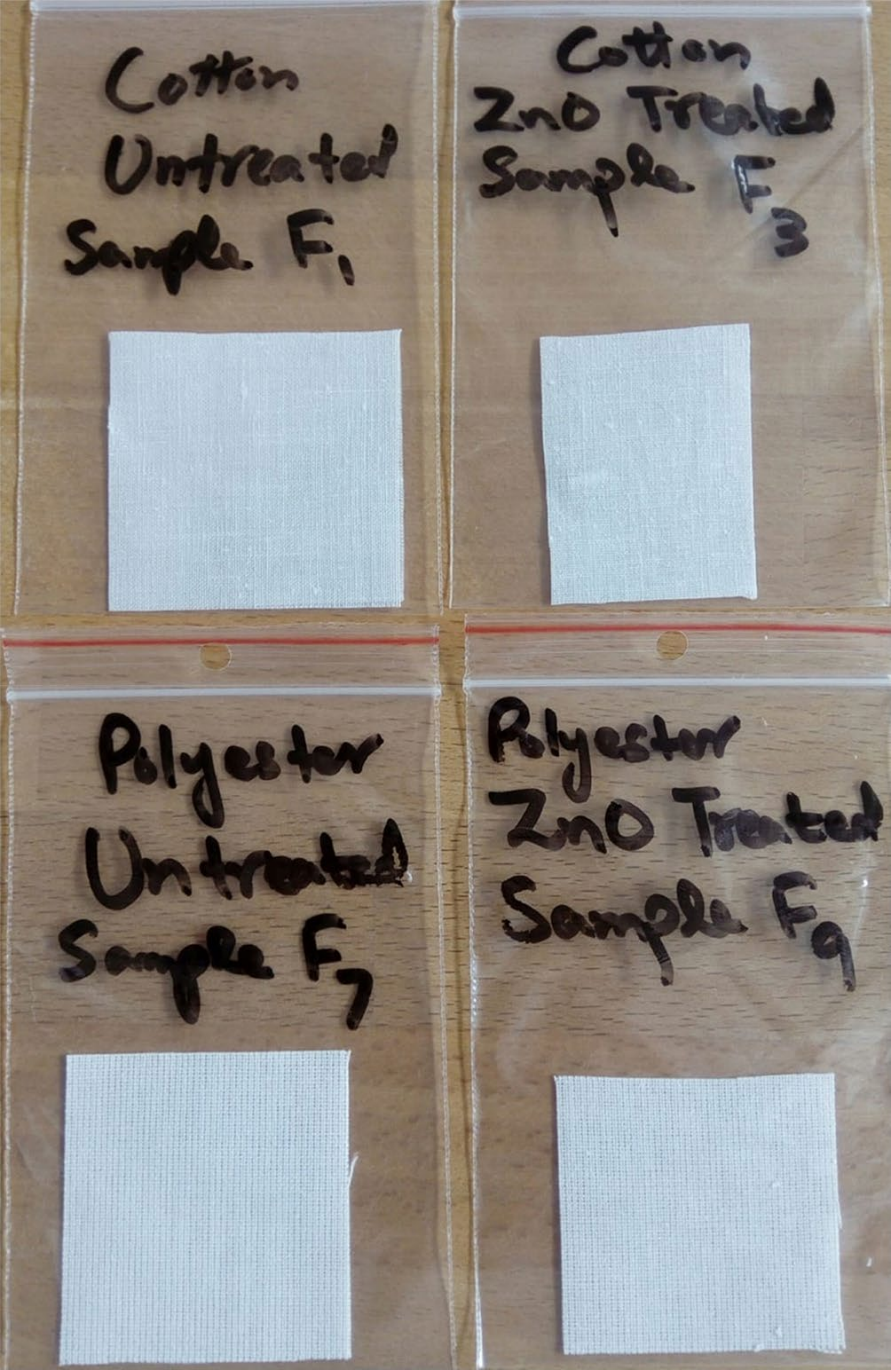
The practical images of untreated cotton and polyester fabrics and ZnO coated cotton and polyester fabrics respectively.

For a twinkling analysis of thermophysiological properties of investigated samples, a spider plot is drawn and shown in Fig. 11. The spider plot is based on original experimental values. The practical images of untreated samples and ZnO treated samples are provided in Fig. 12.

### Washing stability (reusability)

**Figure 13:**
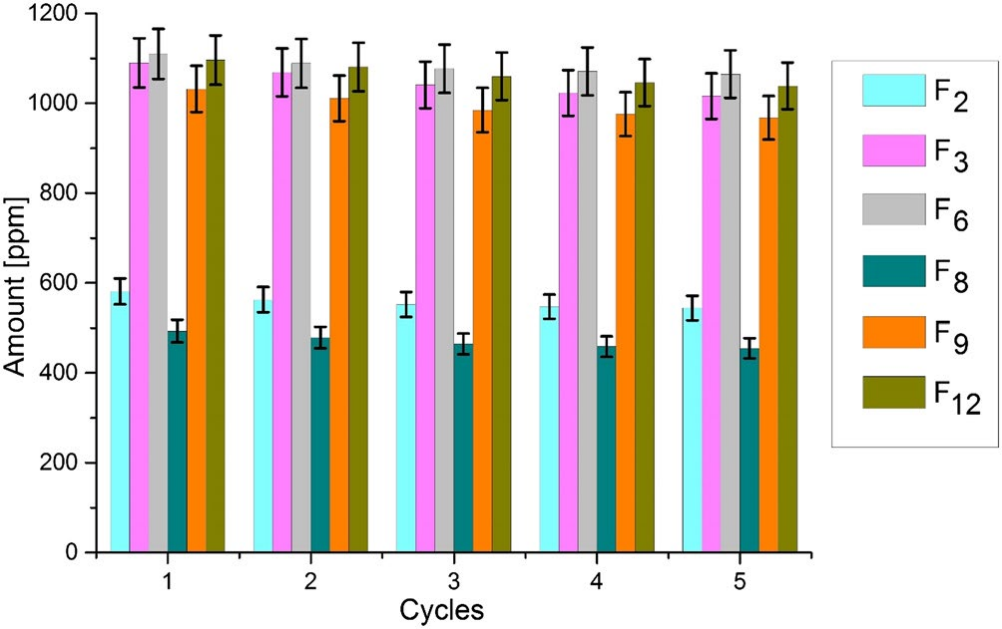
Reusability and washing stability of different samples.

The durability of ZnO NPs deposited on cotton and polyester fabrics was evaluated against washing. In this typical method, total number of Zn^2+^ ions occurred in solution were taken as washing durability. Lower quantity of Zn^2+^ ions indicates higher durability. The total amount of Zn^2+^ ions appeared after 5th cycle was 37 ppm, 74 ppm, 45 ppm, 38 ppm, 64 ppm and 57 ppm for samples F_2_, F_3_, F_6_, F_8_, F_9_ and F_12_ respectively. The observed results are significantly positive and depicts that only 6% ZnO NPs were removed on average from treated samples after 5th cycle whereas sample by sample percentage was 6.3% for F_2_, 6.7% for F_3_, 4.0% for F_6_, 7.7% for F_8_, 6.2% for F_9_ and 5.2% for F_12_ respectively. The reusability results show that ZnO NPs synthesized by sonication were attached adhesively to fabric as their minimal quantity was removed after five consecutive cycles as illustrated in Fig. 13. This confirmed the stability and reusability of synthesized samples.

### Pore size distribution

BET (Brunauer, Emmet and Teller) analysis was performed to estimate pore size distribution and specific surface area. These microstructural properties are highly influential and significantly depends on porosity, geometry and morphology of ZnO NPs. These parameters were determined under N_2_ (− 196 °C) atmosphere. The adsorbed volume of gas on surface of textile substrate was considered as total area including specific surface area and pore size distribution. The representation of experimental conditions and the results of particle size, pore volume, surface area and pore size distribution are reported in Table 3.

**Table 3:** Experimental detail and results of BET analysis of all samples.

Sample ID	Sonication time (min)	ZnO particle size (nm)	Surface area (m^2^ g^−1^)	Pore volume (cm^3^ g^−1^)	Pore size (nm)
F_1_	–	–	–	–	–
F_2_	60	31.6	104	0.31	22
F_3_	60	28.3	113	0.27	26
F_4_	–	–	–	–	–
F_5_	60	30.1	101	0.26	21
F_6_	60	28.4	109	0.22	24
F_7_	–	–	–	–	–
F_8_	60	32.8	110	0.28	23
F_9_	60	29.2	112	0.25	27
F_10_	–	–	–	–	–
F_11_	60	33.1	101	0.24	30
F_12_	60	31.5	104	0.22	32

## Conclusion

The motivation of this work was to evaluate comfort properties of ZnO NPs coated fabrics with varying thickness. The following conclusions were drawn that significantly based on heat and moisture transportation and air permeability.

- Fabric thickness is an effective variable that affects comfort properties particularly thermal conductivity and thermal absorptivity. Furthermore, the results between thermal conductivity and thickness were statistically significant with R^2^ value 0.9125. By keeping comfort feeling in mind, the result illustrated that the deposition of ZnO NPs by sonication improved thermal conductivity significantly. Additionally, the values of thermal conductivity of polyester were higher than cotton in a parallel comparison of thickness.
- Consistent results were found for thermal absorptivity of untreated and treated samples. Fabric thickness played a metaphorical role for thermal absorptivity. For thermal absorptivity, the results of regression analysis showed that the value of R^2^ is a little bit lower as an abnormal distribution was observed. Moreover, the results showed that ZnO NPs coating on fabrics by sonication improved thermal absorptivity to some extent.
- Structure and morphology of textile materials play a major role in the evaluation of fabric comfort. Moisture transportation phenomenon significantly depends on porosity. Relative water vapor permeability results were decreased for both cotton and polyester as fabric thickness and quantity of ZnO NPs increased. This decrease was due to lower porosity.
- Absolute evaporative resistance was increased as thickness and amount of ZnO NPs increased. However, a diminution for absolute evaporative resistance results was observed after ZnO NPs coating on fabrics by sonication. The lateral was sonication impact as ultrasonic energy untie fibre structure and allow fluid to pass through.
- Besides the inspirational findings, many influential variables of heat and moisture transportation i.e. thermal diffusivity, thermal resistance, heat flux, wetting, accumulative one-way transport index etc. still need to investigate because comfort properties could depend on them also. Therefore, for more deeper comfort zone, these variables will be investigated.
